# Osseous Union of Two Supenumerary Teeth

**Published:** 1857-07

**Authors:** 


*Osseous Union of two Supenumerary Teeth.*—We have frequently had occasion to record examples of osseous union of temporary, and several times, of permanent teeth, and quite recently two supernumerary teeth, taken from between and behind the central incisors of the upper jaw, the roots and about one half of the crowns of which are perfectly united, were presented to the senior editor. As all deviations from normal development in the teeth are interesting, at least in a physiological point of view, we take pleasure in mentioning the one under consideration.

